# Probiotic potential of *Saccharomyces cerevisiae* GILA with alleviating intestinal inflammation in a dextran sulfate sodium induced colitis mouse model

**DOI:** 10.1038/s41598-023-33958-7

**Published:** 2023-04-24

**Authors:** Bum Ju Kil, Young Jin Pyung, Hyunjoon Park, Jun-Won Kang, Cheol-Heui Yun, Chul Sung Huh

**Affiliations:** 1 Biomodulation Major, and Center for Food and Bioconvergence, Seoul National University, Seoul, 08826 Republic of Korea; 2 Department of Agricultural Biotechnology, and Research Institute of Agriculture and Life Sciences, Seoul National University, Seoul, 08826 Republic of Korea; 3 Research Institute of Eco-Friendly Livestock Science, Institute of Green-Bio Science and Technology, Seoul National University, Pyeongchang-gun, 25354 Republic of Korea; 4 Department of Food Science and Biotechnology, Dongguk University-Seoul, 32, Dongguk-ro, Ilsandong-gu, Goyang-si, Gyeonggi-do 10326 Republic of Korea; 5 Graduate School of International Agricultural Technology, Seoul National University, Pyeongchang-gun, 25354 Republic of Korea

**Keywords:** Biotechnology, Microbiology

## Abstract

Recently, several probiotic products have been developed; however, most probiotic applications focused on prokaryotic bacteria whereas eukaryotic probiotics have received little attention. *Saccharomyces cerevisiae* yeast strains are eukaryotes notable for their fermentation and functional food applications. The present study investigated the novel yeast strains isolated from Korean fermented beverages and examined their potential probiotic characteristics. We investigated seven strains among 100 isolates with probiotic characteristics further. The strains have capabilities such as auto-aggregation tendency, co-aggregation with a pathogen, hydrophobicity with *n*-hexadecane,1,1-diphenyl-2-picrylhydrazyl scavenging effect, survival in simulated gastrointestinal tract conditions and the adhesion ability of the strains to the Caco-2 cells. Furthermore, all the strains contained high cell wall glucan content, a polysaccharide with immunological effects. Internal transcribed spacer sequencing identified the *Saccharomyces* strains selected in the present study as probiotics. To examine the effects of alleviating inflammation in cells, nitric oxide generation in raw 264.7 cells with *S. cerevisiae* showed that *S. cerevisiae* GILA could be a potential probiotic strain able to alleviate inflammation. Three probiotics of *S. cerevisiae* GILA strains were chosen by in vivo screening with a dextran sulfate sodium-induced colitis murine model. In particular, GILA 118 down-regulates neutrophil–lymphocyte ratio and myeloperoxidase in mice treated with DSS. The expression levels of genes encoding tight junction proteins in the colon were upregulated, cytokine interleukin-10 was significantly increased, and tumor necrosis factor-α was reduced in the serum.

## Introduction

Intestinal inflammation, such as inflammatory bowel disease (IBD), is a chronic relapsing inflammatory disorder of the gastrointestinal tract (GIT)^1^. The most common drugs used for the treatment of inflammatory bowel disease include sulfasalazine, mesalamine (Asacol, Pentasa, Colazal, and Salofalk), azathioprine, 6-mercaptopurine, cyclosporine, infliximab (Remicade), adalimumab (Humira), and corticosteroids (prednisone); however, long-term drug therapy should be avoided to evade their side effects^2^. A common alternative treatment is probiotics. Probiotic research including product development has received increasing attention^3–5^. Probiotics are active microorganisms that improve health and prevent illness^6^. *Lactobacillus* and *Bifidobacterium* are present in most probiotics, especially lactic acid bacteria (LAB) in the pharmaceutical industry^7,8^. It has been suggested that *Saccharomyces boulardii*, a well-known probiotic, can be effective in IBD by inhibiting the Nuclear factor kappa-light-chain-enhancer of activated B cells (NF-κb) signaling pathway^9^. In particular, probiotic research on *S. boulardii* has indicated positive outcomes in treating environment-related guts and anti-inflammatory effects^10,11^.

Prokaryotic probiotics have been studied more extensively than eukaryotic probiotics, and the differences between the prokaryotic (bacteria) and eukaryotic (yeast) probiotics lie in their sizes, cell wall composition, and optimal growth conditions^12^. Yeasts are ten times larger than bacteria, and their cell wall comprises chitin, glucan, mannose, phosphopeptidomannan, and phospholipomannan. Glucan, a cell wall component, modulates the immune system to improve immune functions^13^, presenting a benefit for probiotic applications. Eukaryotic (yeast) probiotics can tolerate stress, survive, and adhere to the gastrointestinal tract. However, yeast application as a probiotic is limited while bacteria use a wide range of animals^12^. Therefore, we need to provide insights into the yeast strain that will benefit the host.

Similar to other microorganisms, the principal function of yeast is fermentation. For instance, *S. cerevisiae* converts glucose to ethanol and carbon dioxide in traditional Korean rice wines such as Makgeolli and Dongdongju, contributing to their characteristic properties^14^. Yeast also protects rice wine from contamination by other bacteria^14^. Some yeast strains also have high nutrient value^15^, and the ability of yeast to change cereals to fermented and functional foods is closely related to health issue^16^. Because of their safety and technological applications, *S. cerevisiae* species have been extensively studied and used in various sectors, including bakeries and breweries. Several reports have demonstrated and documented the benefits of *S. cerevisiae* strains on human health^17–19^. Functions of *S. cerevisiae*’s including its anti-infective properties^17^, antioxidant activities^18^, and other probiotic properties, have also been studied. Recently, yeast *S. cerevisiae* UFMG A-905 showed probiotic properties when treated in mice infected by *Salmonella Typhimurium*^20^. These findings suggest that the probiotic *S. cerevisiae* varieties should be carefully selected for the optimum host health. Therefore, screening of probiotic *S. cerevisiae* is of utmost importance, and our findings highlight the probiotic potential of *S. cerevisiae*.

The objective of the present study, we demonstrated the probiotic potential of *S. cerevisiae* strains derived from Korean rice wine. The Immunomodulatory activity of *S. cerevisiae* was compared with *S. boulardii* to determine nitric oxide production by RAW 264.7 cells. Furthermore, in vivo studies were done to choose the *S. cerevisiae* GILA stain that alleviated intestinal inflammation functionality in a DSS-induced colitis model.

## Results

### Tolerance to in vitro GI tract conditions

Yeast cell growth was less resistant at pH 2.0 than *S. boulardii* CNCM I-745. We only chose similar or higher growth yeast than *S. boulardii* CNCM I-745. Table 1 shows the results for identified yeast strains. According to resistance to pH 2.0 and bile condition, test *S. cerevisiae* GILA strain showed a similar survival rate. *S. cerevisiae* were chosen to compare the growth in the bile condition with *S. boulardii* CNCM I-745. *S. boulardii* control strains had almost 100% survivability in 3.0% Oxgall. The strains with over 90% survivability were selected, where most strains showed > 90% survivability.

**Table 1:** Percentage of acid tolerance and bile tolerance.

*S. cerevisiae* strains	Acid tolerance (%)	Bile tolerance (%)
*S.b* CNCM I-745	99.53 ± 0.69	97.85 ± 0.44
*S.c* GILA 59	97.06 ± 1.34	104.4 ± 0.63
*S.c* GILA 100	96.46 ± 2.42	99.64 ± 1.65
*S.c* GILA 106	101.66 ± 1.24	98.66 ± 0.50
*S.c* GILA 115	100.85 ± 0.37	100.12 ± 2.00
*S.c* GILA 118	99.19 ± 1.36	98.25 ± 2.01
*S.c* GILA 137	98.21 ± 0.32	97.98 ± 1.23
*S.c* GILA 197	99.99 ± 1.08	95.31 ± 3.32

**Figure 1:**
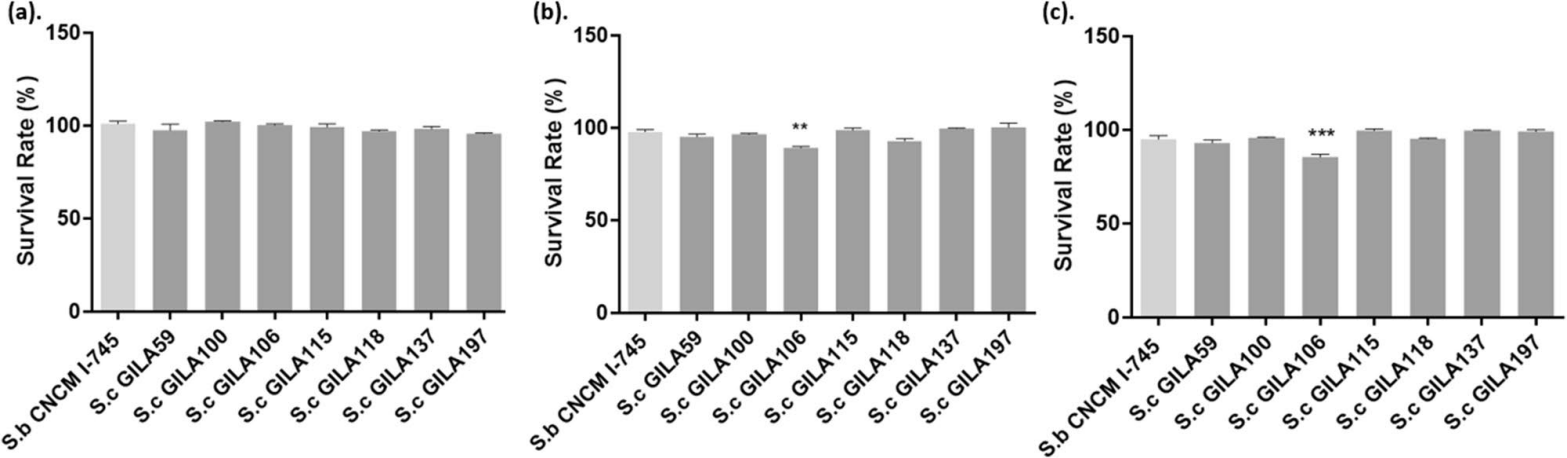
The survival rate of *S.cerevisiae* strain at (**a**) mouth, (**b**) stomach and (**c**) intestine in the GIT model. Values are mean ± S.D of triplicates for each group. **p < 0.01, ***p < 0.001, compared with *S. boulardii* CNCM I-745.

The survival rates of all *S. cerevisiae* GILA strains were similar when compared with control strain *S. boulardii,* CNCM I-745, which showed over 95% survival in GIT model at mouth, stomach and intestine (Fig. 1). *S. cerevisiae* GILA106 showed a significantly lower survival rate (*p* < 0.01 and *p* < 0.001) than other *S. cerevisiae* GILA strain in the stomach and intestine, whereas all strains showed about a 90.0% survival rate.

### Aggregation and adhesion properties to caco-2 cell

Coaggregation with pathogen was high in *S. boulardii* CNCM I-745. We selected *S. cerevisiae* GILA stains with more significant or similar to coaggregation ability than *S. boulardii* CNCM I-745 (Table 2)*.* Most of the isolated yeasts showed high autoaggregation properties. Aggregation characteristics of yeast are related to sporulation^21^. Notably, 80% autoaggregation after 24 h of incubation was based on colony formation^22^, which affects host colonization after entry. Those strains with autoaggregation ability could also have coaggregation ability with the pathogen. *S. boulardii* CNCM I-745 had between 60 and 85% coaggregation with the three pathogens, *Staphylococcus aureus* ATCC 25922, *Enterococcus faecalis* ATCC 29212 and *Escherichia coli* K88.

**Table 2:** Percentage of autoaggregation and coaggregation with pathogens.

*S. cerevisiae* strains	Coaggregation (%)			
	Autoaggregation (%)	*S. aureus* ATCC 25922	*E. faecalis* ATCC 29212	*E. coli* K88
*S.b* CNCM I-745	88.65 ± 1.82^ab^	71.04 ± 28.66^a^	60.31 ± 17.07^bc^	68.07 ± 11.78^a^
*S.c* GILA 59	97.97 ± 2.19^a^	49.42 ± 23.97^ab^	62.39 ± 37.90^abc^	55.75 ± 5.30^abc^
*S.c* GILA 100	97.39 ± 1.00^a^	60.30 ± 29.30^ab^	50.37 ± 12.76^c^	44.25 ± 33.50^abc^
*S.c* GILA 106	84.85 ± 14.94^b^	74.79 ± 8.12^a^	97.65 ± 7.15^a^	57.74 ± 0.57^ab^
*S.c* GILA 115	99.09 ± 0.44^a^	66.50 ± 4.42^ab^	41.18 ± 7.69^bc^	47.69 ± 7.73^abc^
*S.c* GILA 118	90.32 ± 3.55^ab^	73.16 ± 21.88^ab^	47.41 ± 9.10^c^	23.23 ± 1.43^c^
*S.c* GILA 137	91.72 ± 5.67^ab^	22.79 ± 0.97^b^	89.38 ± 7.39^ab^	24.79 ± 14.19^bc^
*S.c* GILA 197	95.81 ± 5.83^ab^	61.56 ± 3.30^ab^	69.63 ± 14.83^abc^	49.39 ± 7.58^abc^

Mean values in the same column with different superscript letters are significantly different (*p* < 0.05).

**Figure 2:**
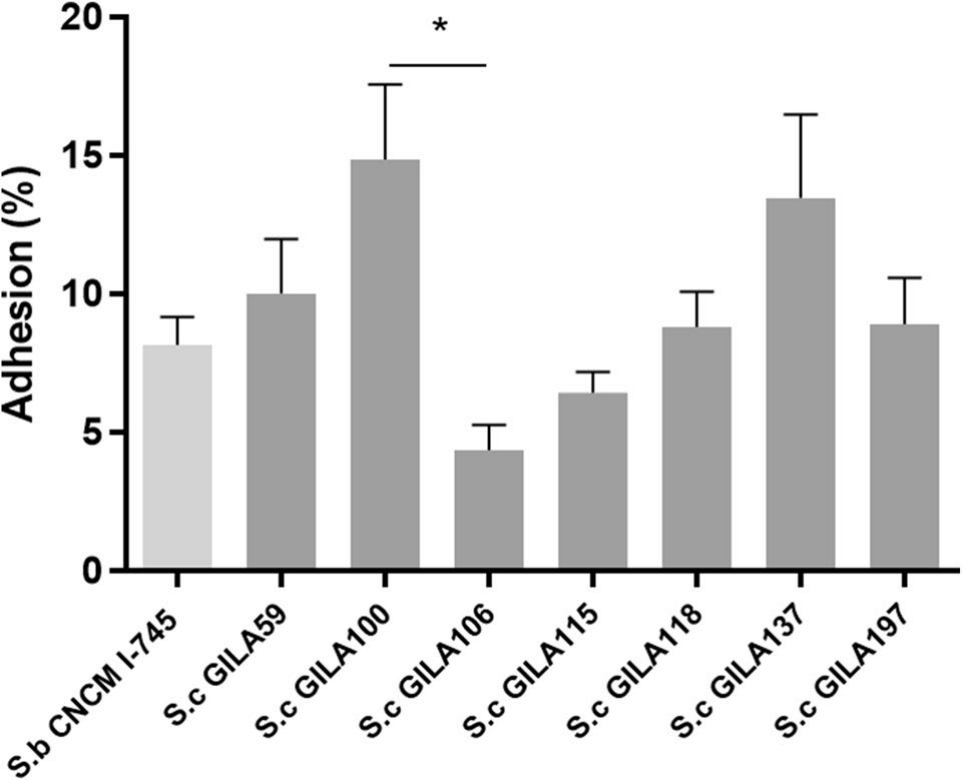
Adhesion percentages of *S.cerevisiae* strain on caco-2 cells. Adhesion percentages are calculated by plate count method. Mean values in the same column with different superscript letters are significantly different (*p < 0.05).

Adhesion assay to Caco-2 cell show all *S. cerevisiae* GILA strain’s adhesion ability (4% to 15%). When compared with the control yeast strain, *S. boulardii* CNCM I-745, *S. cerevisiae* GILA 100, 118, 137, 197 were not significantly (*p* > 0.05) different. *S. cerevisiae* GILA 106 was significantly (*p* < 0.05) lower than GILA 100 (Fig. 2).

### Antioxidant ability by DPPH assay

**Figure 3:**
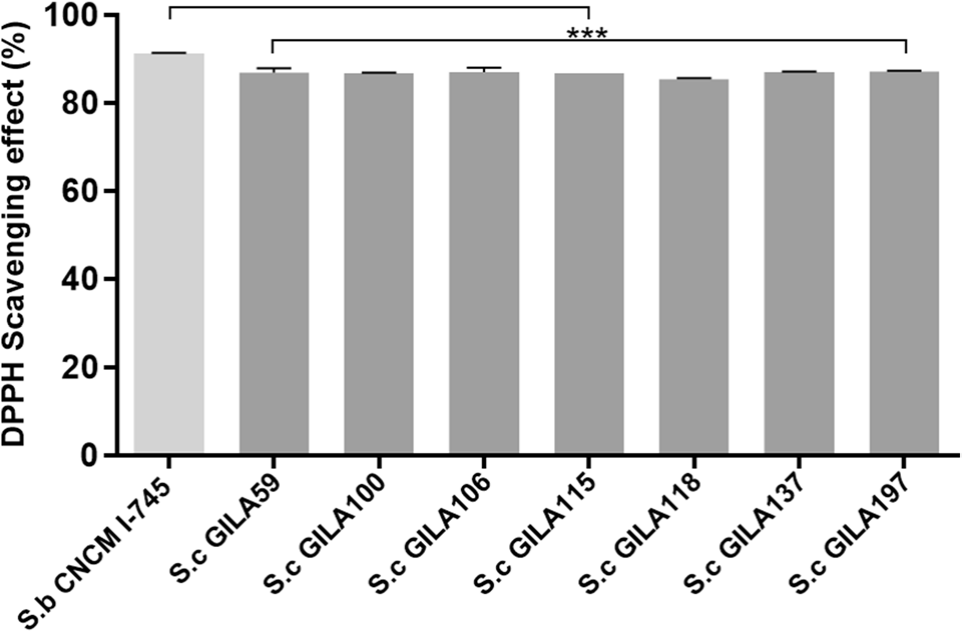
Screening of *S.cerevisiae* with over 85% DPPH scavenging effect. After 30 min incubation, the absorbance was converted to the scavenging effect (%). Values are mean ± S.D of triplicates for each group. ***p < 0.001, compared with *S. boulardii* CNCM I-745.

The DPPH scavenging effect of *S. cerevisiae* GILA strains was evaluated to examine their antioxidant ability. The scavenging results for *S. boulardii* CNCM I-745 were 91.35 ± 0.13%; results for other yeasts were less than 90.00%. For this reason, we chose those strains with more than 85.00% activity (Fig. 3). In the final stage of probiotic research, we compared these results with cell wall β-glucan.

### Phylogenetic analysis of the ITS region

**Figure 4:**
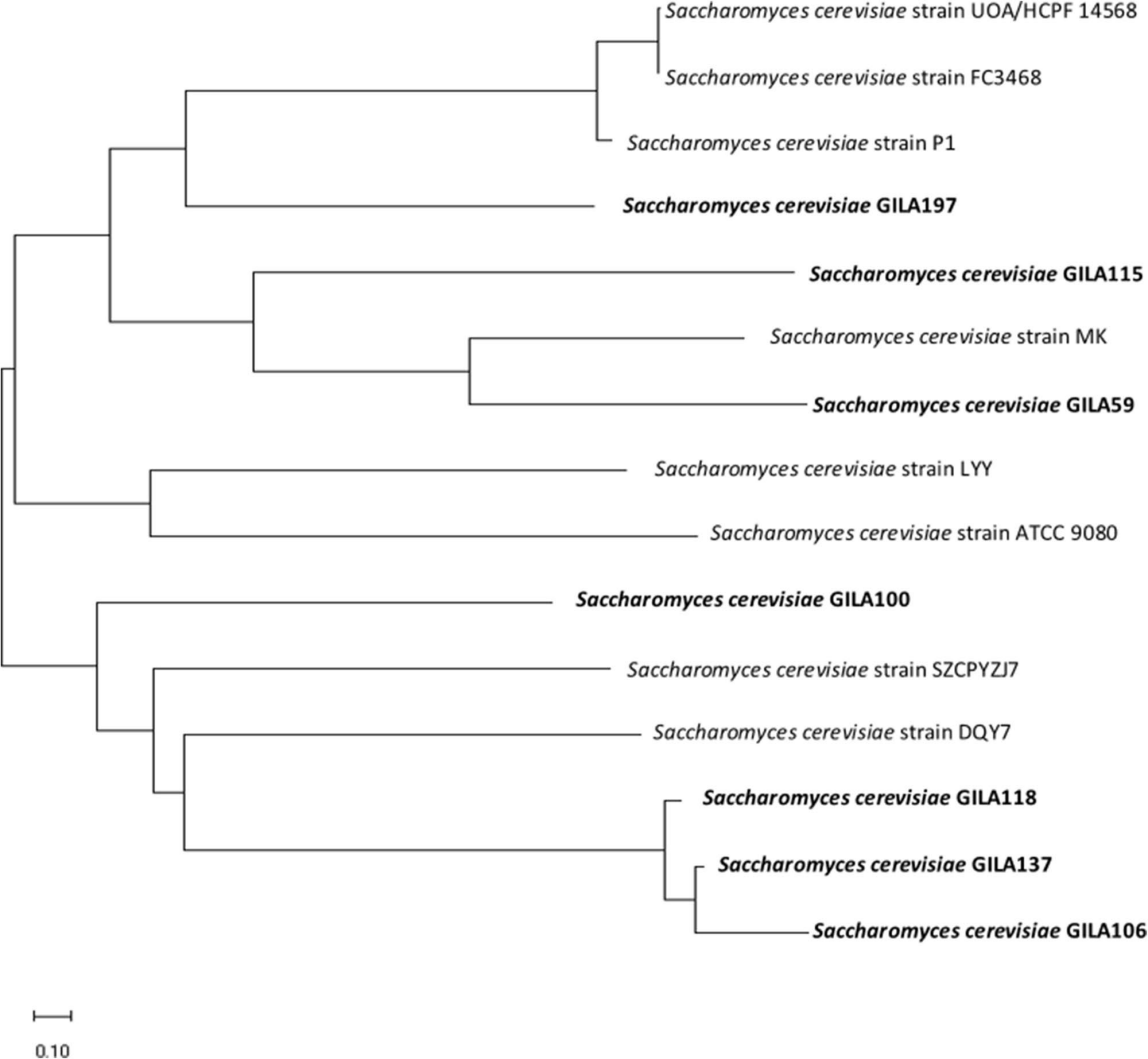
The phylogenetic tree of *S. cerevisiae* GILA. Based on ITS region sequencing. Relationships of taxa was inferred using the Neighbor-Joining method. Bar, 0.10 substitutions per nucleotide position.

High homology of ITS sequences was observed closest location of *S. cerevisiae* GILA 137 to *S. cerevisiae* GILA 106 and closer to *S. cerevisiae* GILA 118 in the 15 yeasts (Fig. 4). *S. cerevisiae* GILA 100 is closely related to *S. cerevisiae* GILA 106, 118, and 137. Due to the high homology of *S. cerevisiae* GILA, phenotypic differences such as the probiotics potential of *S. cerevisiae* GILA were investigated.

### Alleviating inflammation in 264.7 cells and splenocyte

**Figure 5:**
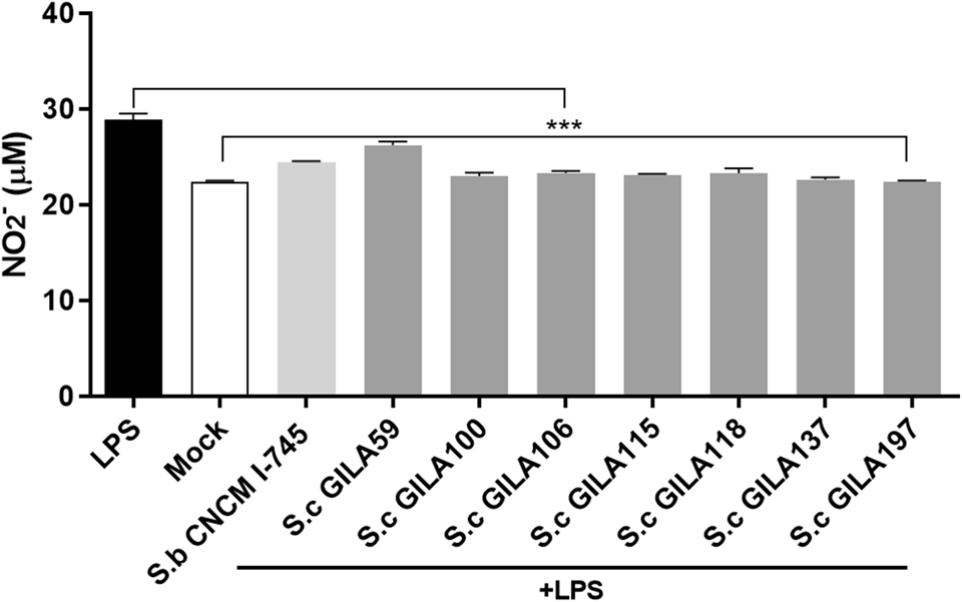
Nitric oxide production of heat-killed *S.boulardii* and *S.cerevisiae* strains in LPS (1 µg/ml) induced RAW 264.7 murine macrophage cells. The concentration of Nitric oxide production was determined by calculating standard curve. Values are mean ± S.D of triplicates for each group. ***p < 0.001, compared with treatment of only LPS.

NO is related to various immunological procedures such as host defense, immunoregulation and signal transduction and are importance mediators triggering gastrointestinal disease^23^. NO is produced from L-arginine by an enzyme of nitric oxide synthase (NOS) and the inducible isoform of NO (iNOS) during inflammation where iNOS is activated by pro-inflammatory cytokines like tumor necrosis factor-α (TNF-α), interleukin-6 (IL-6). For examining the effects of selected *S. cerevisiae* GILA, 10 ng/ml of LPS was treated in 264.7 cells for 48 h to induce inflammation and NO production. Selected *S. cerevisiae* GILA significantly (*p* < 0.001) suppressed NO production induced by LPS compared with the positive control treated LPS only. *S. boulardii* CNCM I-745 has shown anti-inflammatory effects compared with the LPS treatment group (Fig. 5).

### Alleviating the intestinal inflammation in a dextran sulfate sodium-induced colitis mouse model

**Figure 6:**
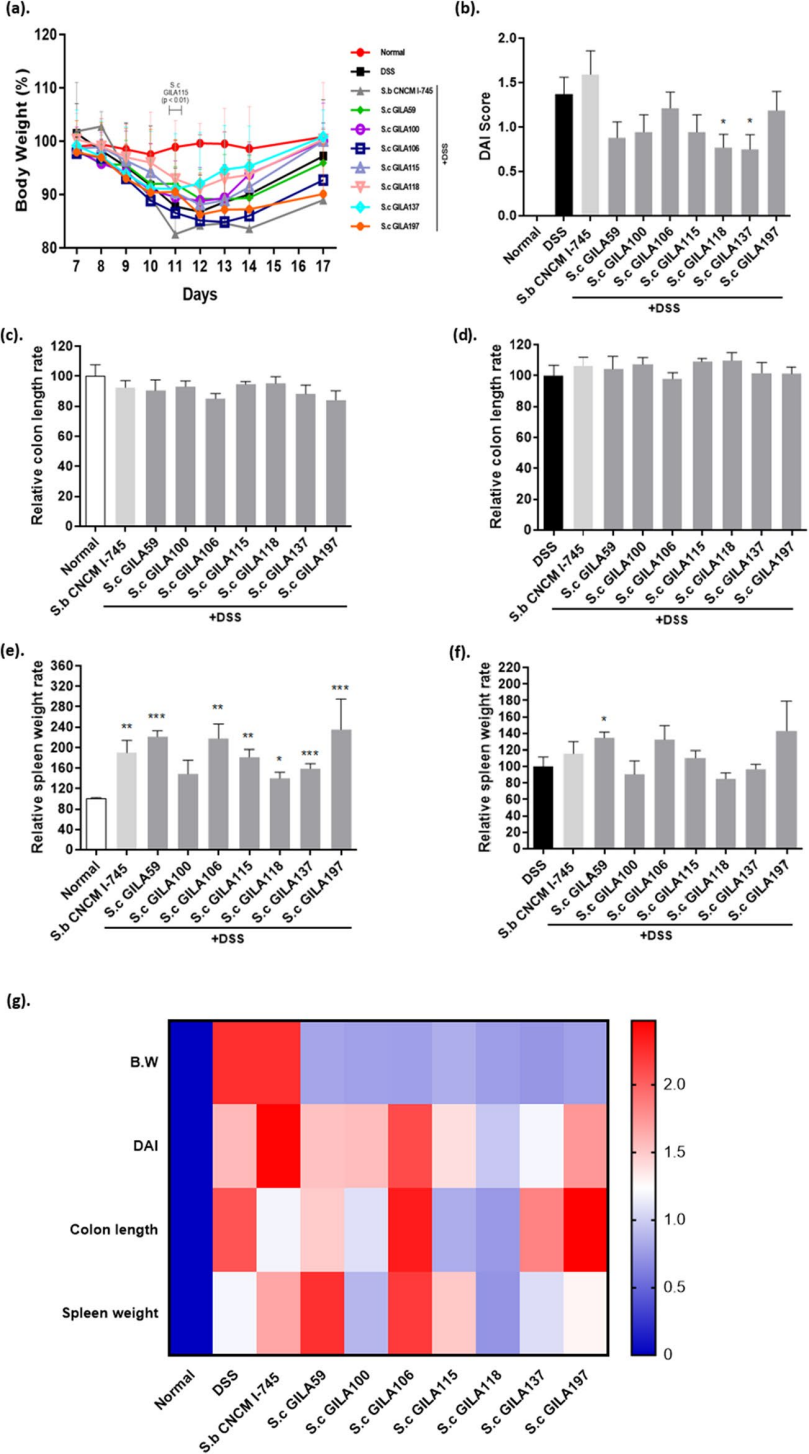
In vivo screening of *S.cerevisiae* GILA strain. Pathological and physiological status through the indicators of inflammation. (**a**) Body weight (%) compared to the normal group. (**b**) DAI score of disease from C57BL/6 J mouse group. Relative colon length rate compared with that of Normal (**c**) and DSS (**d**) group. Relative spleen weight rate compared with that of Normal (**e**) and DSS (**f**) group. (**g**) S.c GILA strain’s in vivo screening total score. Statical significance is indicated as follows: *p < 0.05, **p < 0.01 and ***p < 0.001.

During DSS treatment with yeast period, the *S. cerevisiae* GILA115 group showed body weight loss more than the normal group (*p* < 0.01) on day11. In contrast the other *S. cerevisiae* GILA groups were not significantly (*p* > 0.05) different (Fig. 6a). Stool consistency and bleeding score were significantly lower in the *S. cerevisiae* GILA 59, 100, 118, and 137 groups than in the DSS group, although *S. cerevisiae* GILA 59 and 100 groups lose more weight (8–10%) than *S. cerevisiae* GILA 118 and 137 groups (4–8%). Consequently, *S. cerevisiae* GILA 118 and 137 groups were significantly (*p* < 0.05) lower than the DSS treatment group when compared with the disease activity index (DAI) score (Fig. 6b). The relative colon length rate was not significantly different (Fig. 6c,d) whereas the relative spleen weight rate was significantly different between the normal and DSS treatment groups (Fig. 6e,f). *S. cerevisiae* GILA100 and 118 groups were similar spleen weight rates to the normal group (Fig. 6e). Standard scores were calculated using normal and DSS group scores. The total score showed GILA 100, 118, and 137 groups were similar to the normal group than the other groups (Fig. 6g).

**Figure 7:**
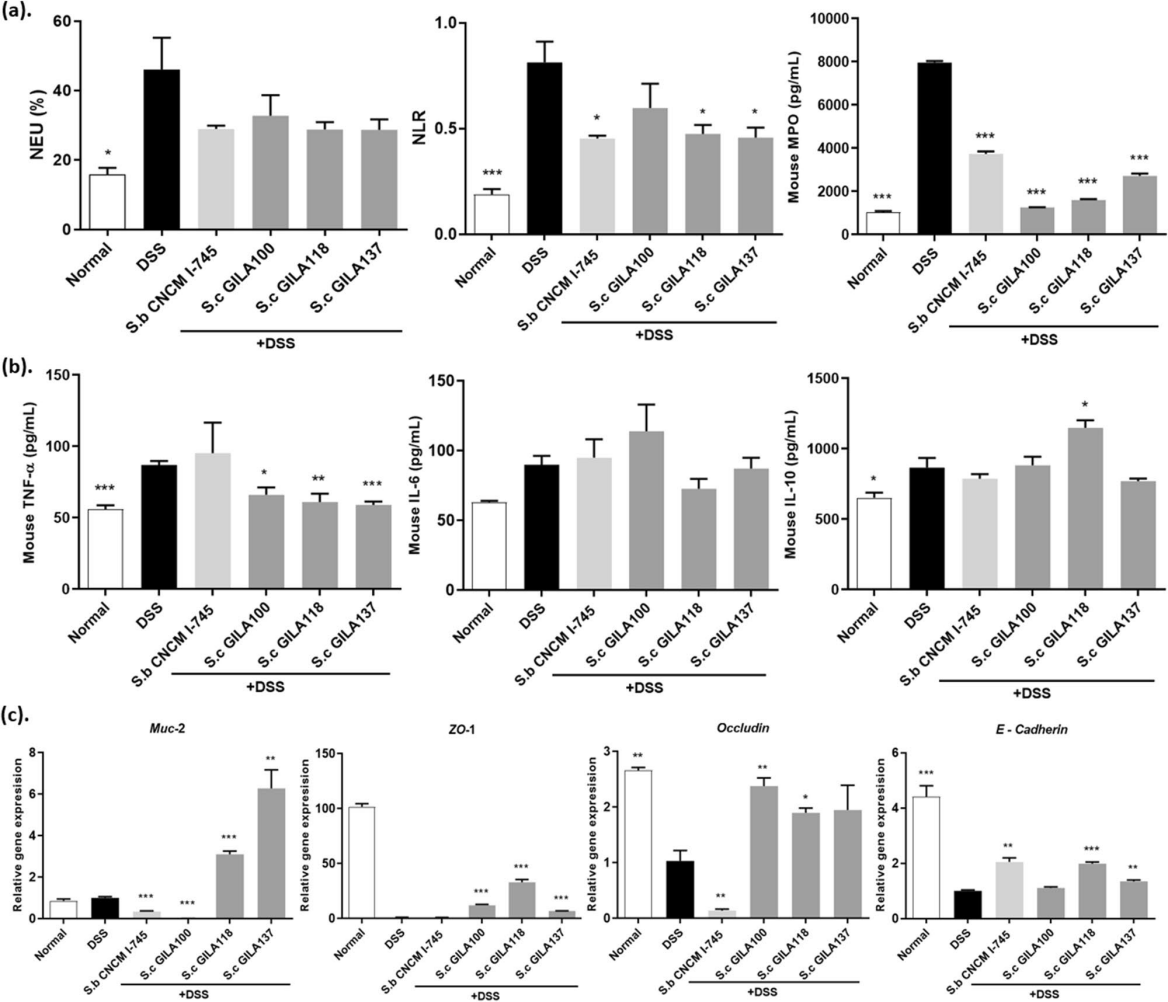
Alleviation of Intestinal Inflammation in Mice between Treatments of *S. cerevisiae* GILA. (**a**) Neutrophil, Neutrophil–lymphocyte ratio from complete blood cell count and Amount of MPO in feces. (**b**) Analysis of pro-inflammatory cytokine TNF-α, IL-6 and anti-inflammatory cytokine IL-10 in serum. (**c**) Relaive gene expression of Muc-2, ZO-1, Occludin, E-cadherin in colon tissue. Statical significance is indicated as follows: *p < 0.05, **p < 0.01, ***p < 0.001.

To investigate the therapeutic properties of *S. cerevisiae* GILA strains in vivo, the DSS group showed IBD-colitis symptoms, including increased neutrophil count, neutrophil–lymphocyte ratio (NLR) in blood, myeloperoxidase (MPO) in feces (Fig. 7a), and proinflammatory cytokine (TNF-α) in serum (Fig. 7b). Stool consistency and bleeding score results were related to NLR from complete blood cell count (Fig. 7a)^24,25^. This finding suggested that the DSS-induced increase in neutrophils may affect other biomarkers and cytokines. Neutrophil expression in blood is one of the main features of colitis^26–28^. Further analysis was conducted to investigate the amelioration of intestinal inflammation. The gene expression levels of mucin-2 (Muc-2), zonula occludens-1 (ZO-1), occludin and epithelial cadherin (E-cadherin) significantly increased compared with those in the DSS group. (Fig. 7c).

We found that the *S. cerevisiae* GILA group had significantly decreased NLR in the blood (*p* < 0.01), MPO in feces (*p* < 0.001), and TNF-α in serum (*p* < 0.05*, p* < 0.01 and* p* < 0.001) (Fig. 7a,b). No significant changes were observed for IL-6 in serum. Meanwhile, IL-10 in serum significantly increased in the *S. cerevisiae* GILA 118 group compared with that in the other groups. The *S. cerevisiae* GILA 118 group also showed significantly increased serum IL-10 levels compared with the DSS group (Fig. 7b). These results suggested that *S. cerevisiae* GILA 118 effectively inhibits the biomarker of IBD and the expression of IL-10, thereby ameliorating colitis in mice.

## Discussion

In our investigation, we selected a probiotic candidate when we applied *S. cerevisiae* GILA, which had a high survival rate in low pH and bile conditions. These results indicate that yeast which had high resistance to harsh condition also had high survival in GIT simulation model. *S. cerevisiae* GILA has almost over 90.0% survival rate in GIT model. Tolerance to GI tract could be considered in vivo conditions. Comparing the viable counts in the murine gastrointestinal tract could be shown survival rate *in vivo*^29^. Survival was an important factor of probiotics, but safety was also studied for selected probiotic potential yeast. None of the selected *S. cerevisiae* GILA strain had hemolytic activity and biogenic amine production. Yeast is also known to have antibiotic resistance. Yeasts aggregate via their cell wall mannose^30^; a prominent aggregation ability indicates ample mannose. *S. cerevisiae* GILA stain showed 90% hydrophobicity (data not shown). The adhesion ability for Caco-2 cells, such as microbial adhesion to mucosa model^31^ was 4–15%. These results show that *S. cerevisiae* GILA stain possesses cell adhesion ability to be used in probiotic preparation.

The DPPH scavenging effect measures the antioxidant capacity related to the cell wall β-glucan content. β-Glucan is a β-d-glucose polysaccharide group and a component of the yeast cell wall. Its structure is a long, β-(1,6)-branched, β-(1,3)-glucan^32^. β-Glucan has an excellent antioxidant capacity^33^; however, this could not explain the results for cell wall β-glucan (Fig. S1). Yeast itself may then possess an intrinsic antioxidant ability^34^. Therefore, there should be another experiment to quantify cell wall β-glucan. β-Glucan is recognized by the receptors on the host’s immune cells^35^, enhancing immune function resulting in anticancer and anti-inflammatory effects^35,36^. Furthermore, yeast β-Glucan benefits host health by protecting it against pathogens^37^. The content of β-Glucan was calculated from total glucan by subtracting α-glucan. A large amount of β-glucan in the yeast cell walls was comparable to a previous study^38^. The *S. cerevisiae* GILA strain had more than 36% β-glucan; this result could imply a probiotic potential (Fig. S1). Commercial probiotics, such as *Lactobacillus rhamnosus* GG have 5% β-glucan (data not shown), respectively. *S. cerevisiae* has more than triple the amount of β-glucan than these *Lactobacillus* strains. Moreover, the structure of β-glucan should also be considered in future research. Since β-glucan modulates cytokines in human blood, it should be confirmed whether all structures of β-glucan are beneficial to host health^39^. This result indicated that β-glucan benefits the host’s health and immune system. A future probiotic approach could consider yeast’s β-glucan characteristics. Our results revealed the quantity of β-glucan in the yeast cell walls. This method could be applied during probiotics-related yeast and β-glucan screening. The result was unexpected from the DPPH scavenging effect, but all experimental strains had more than 36% β-glucan, which is a sufficient level^38^.

The cell wall had more than 36% β-glucan, and nitric oxide production was significantly lower than the control. The β-glucan quantity could be edequate, but we need more evidence of related probiotic functionality. Previous studies have reported the prevention of inflammation by yeast fermentate^40^; there are also reports on β-glucan-mediated induction of proinflammatory cytokines^41^. Given that the quantity of β-glucan did not influence this outcome, another factor, such as the probiotic properties of the *S. cerevisiae* GILA strain, may have been responsible for reducing in proinflammatory cytokines. The physiological impact of yeast probiotics against the host was determined by their ability to relieve oxidative stress measured by fecal MPO level^42^. Activated neutrophils release MPO, a marker of oxidative stress, and destroy epithelial cells^26^. As demonstrated by DPPH scavenging capacity of *Saccharomyces cerevisiae* GILA in vitro (Fig. 3), in vivo results also prove this ability to relieve oxidative stress (Fig. 7a). The *S. cerevisiae* strain was resistant to ETEC infection^17^, and *S. cerevisiae* cell wall glucan had an immune-modulatory effect, which could affect colitis reduction^13^. Spleen weight could be due to alleviating intestinal immune response^43^. Moreover, the inflammation biomarkers were similar to those in the CBC test-neutrophil lymphocyte rate results^28^ (Fig. 7a,b). There was no significant difference in the relative expression rate of the Muc-2 gene between the normal and DSS groups (Fig. 7c). Recovery through a 6-days period (Fig. S2) is considered maintaining in the DSS group. In this study, an increase in Muc-2 gene expression was regarded as an improved capacity of epithelial protection^44^. As a result, similar to this experiment, colitis was alleviated by increasing Muc-2 expression in intestinal goblet cells^45^. An increase in Muc-2 gene expression is assumed to reduce colitis (Fig. 7c). Furthermore, we elucidated that anti-inflammatory cytokine IL-10 in serum was more upregulated by *S. cerevisiae* GILA 118 than other *S. cerevisiae* GILA strains. These findings warrant further experiments, especially *S. cerevisiae* GILA 118 structure study with the DSS-colitis model. For this reason, *S. cerevisiae* could be developed as a useful probiotic in the future. Nevertheless, more evidence as a potential gut microbiota modulator^46^ is required for the *S. cerevisiae* GILA strain-related probiotics. Thus, further additional research and development are required to characterize these probiotic candidates' functionality.

## Conclusion

**Figure 8:**
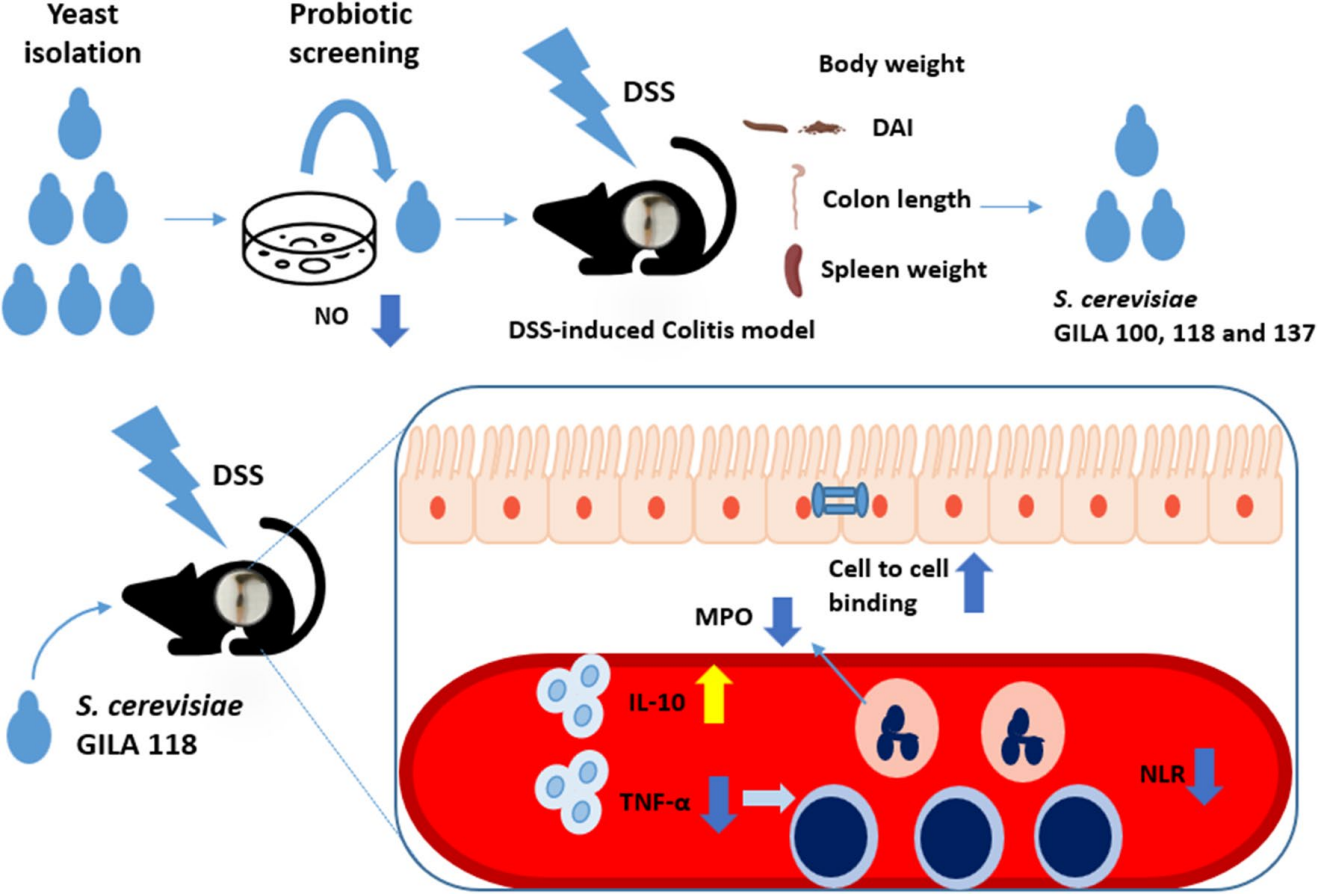
Graphical summary. Isolation of probiotic potential *S. cerevisiae* GILA in Korean rice wine and *S. cerevisiae* GILA118 met the criteria for a probiotic which had alleviating inflammatory effect.

We screened and selected seven *S. cerevisiae* strains as probiotic properties were similar to or higher than *S. boulardii* CNCM I-745. The strain *S. cerevisiae* GILA100, GILA118, and GILA137 met the criteria for a probiotic which had to alleviate inflammatory effect in a DSS-induced colitis mouse, especially *S. cerevisiae* GILA 118 administration increased IL-10 in serum and also alleviated intestinal inflammation in mice compared with *S. cerevisiae* GILA 100 and GILA137 (Fig. 8).

## Materials and methods

### Isolation and culture conditions

Eight samples of rice wine were obtained from Gangwon-do and three from Chungcheong-do, both in Korea. Ninety-two strains of yeast were from makgeolli, and eight strains were from dongdongju. All isolates were confirmed by gram staining and cell morphology^47^.

Yeasts were cultured in yeast extract–peptone–dextrose (YPD) broth, consisting of 1% (w/v) yeast extract, 2% (w/v) peptone, and 2% dextrose. To screening yeasts that aggregate with a pathogen, *Staphylococcus aureus* ATCC 25922, *Enterococcus faecalis* ATCC 29212, and *Escherichia coli* K88 were cultured in brain heart infusion broth.

*Saccharomyces boulardii* CNCM I-745, supplied by Jarrow Formulas (Los Angeles, USA), was used as a control for comparison with the isolated yeast strain. The yeast strains were incubated aerobically at 37℃ for 24 h before use simulating the conditions in a human host^48^. All broth and agar materials were obtained from Difco (USA).

### Resistance to low pH and bile conditions

To determine the ability of yeast strains to survive in GI conditions, the isolates were incubated in YPD broth at 37 °C for 24 h. Then, the cultured yeasts were centrifuged at 5500×*g* for 10 min at 4 °C. The pellets were incubated for 2 h in YPD broth, and adjusted to pH 2.0 with 1 N HCl. The sample (100 μL), diluted in phosphate-buffered saline (PBS), was spread on YPD agar according to the drop-plating method^49^. After incubation in YPD broth (pH 2.0) at 37 °C, the resistance of yeast to bile was estimated similarly. The pellets were incubated for 12 h in YPD broth with 3.0% bovine bile (Oxgall, Difco, USA). The survival rate at pH 2.0 and in 3.0% Oxgall was calculated using the following formula: acid and bile tolerance (%) = [yeast after 24 h incubation (log cfu/mL)/yeast after 2 h incubation at pH 2.0 (log cfu/mL) and in 3.0% Oxgall (log cfu/mL)] × 100^50^.

### Autoaggregation and coaggregation with pathogen

Yeasts were grown for 24 h at 37 °C in YPD broth, then harvested by centrifugation at 5500×*g* for 15 min. The pellets were washed twice with PBS, and then resuspended in PBS. Cell suspensions (4 mL) were mixed by vortexing for 10 s, and autoaggregation was determined after 24 h of incubation at 37 °C. The upper suspension layer (0.1 mL) was transferred to another tube with 3.9 mL PBS, and the absorbance (A) was measured at 600 nm. The autoaggregation percentage was calculated as follows: [1 − (A_24_/A_0_)] × 100^51^.

For the coaggregation test, 2 mL each of yeast and pathogen, *Staphylococcus aureus* ATCC 25922, *Enterococcus faecalis* ATCC 29212, and *Escherichia coli* K88 were vortexed together. The level of coaggregation with pathogen was calculated according to the equation of Handley et al.^52^ as follows: coaggregation (%) = [((Ax + Ay)/2) − A(x + y)/((Ax + Ay)/2)] × 100, where x and y represent the yeast and pathogen in the control tube, respectively, and (x + y) is the mixture.

### Hydrophobicity

The hydrophobicity of the yeast cell surface [H(%)] was estimated using adhesion to *n*-hexadecane (Sigma, USA) according to the method by Rosenberg et al^53^ with a slight modification as follows: H(%) = [(1 − OD_4_)/OD_0_] × 100, where OD is the optical density at 0 and 4 h.

### 1-Diphenyl-2-picrylhydrazyl (DPPH) scavenging effect

The DPPH scavenging assay was performed to compare the antioxidant abilities of the yeast strains. The yeast pellets were harvested, washed twice, and resuspended in 1 mL PBS. The resulting suspensions (800 μL) were added to 1 mL DPPH solution (0.2 mM in 80% methanol) and mixed by vortexing, followed by incubation for 30 min in the dark. After the incubation, the solutions were centrifuged at 12,400×*g* for 5 min, and 300 μL of each sample was transferred to a 96-well plate to measure the absorbance at 517 nm. The reconstitution of the standard was performed by adding ascorbic acid to 80% (v/v) methanol at a concentration of 1 mg/mL to 400 μL/mL. Five twofold serial dilutions were performed, and 80% methanol served as the zero standard^54^.

### In vitro gastrointestinal tract (GIT) models

The constituents and concentrations of the various synthetic juices of the in vitro GIT model are shown in Table 3. All materials were obtained from Sigma-Aldrich (USA) or Difco (USA). The inorganic and organic solutions are mixed with distilled water. The pH of the juices and incubation time are adjusted to human physiological traits with a minor modification^55,56^. 7 ml of the *S. boulardii* CNCM I-745 and *S. cerevisiae* GILA strain were centrifuged, then resuspended in 1 ml PBS. Saliva was added and incubated for 5 min. After the incubation, gastric juice was mixed and incubated for 2 h. 12 ml of duodenum juice with 6 ml of bile juice were mixed and incubated for 2 and 5 h with agitation (60×*g*) at 37 °C. Yeast samples were harvested three times and serially diluted and plated onto YPD agar.

**Table 3:** The solution, mixture, pH for in vitro gastrointestinal tract (GIT) models.

	Inorganic solution	Organic solution	Add to mixture	pH
Saliva (Mouth)	KCl 89.6 g/L	Urea 25 g/L	145 mg α-amylase	6.5 ± 0.2
	KSCN 20 g/L		15 mg uric acid	
	NaH_2_PO_4_ 88.8 g/L		50 mg mucin	
	Na_2_PO_4_ 57 g/L			
	NaCl 175.3 g/L			
	NaOH 40 g/L			
Gastric Juice (Stomach)	NaCl 175.3 g/L	Glucose 65 g/L	1 g BSA	3.2 ± 0.2
	NaH_2_PO_4_ 88.8 g/L	Glucuronic acid 2 g/L	1 g pepsin	
	KCl 89.6 g/L	Urea 25 g/L	3 g mucin	
	CaCl_2_·2H_2_O 22.2 g/L	Glucoseamine		
	NH_4_Cl 30.6 g/L	Hydrochloride 33 g/L		
	HCl 37% g/g			
Duodenal Juice (Intestine)	NaCl 175.3 g/L	Urea 25 g/L	CaCl_2_·2H_2_O 22.2 g/L	7.8 ± 0.2
	NaHCO_3_ 84.7 g/L		1 g BSA	
	KH_2_PO_4_ 8 g/L		3 g pancreatin	
	KCl 89.6 g/L		0.5 g lipase	
	MgCl_2_ 5 g/L			
	HCl 37% g/g			
Bile Juice (Intestine)	NaHCO_3_ 84.7 g/L	Urea 25 g/L	CaCl_2_·2H_2_O 22.2 g/L	8.0 ± 0.2
	KCl 89.6 g/L		1.8 g BSA	
	HCl 37% g/g		6 g bile (chicken)	

### Adhesion assay

The Caco-2 cell line, obtained from the Korea cell line bank (KCLB, Seoul, Korea), was used between passages 40–60 for all experiments. The cells were maintained in Dulbecco’s Modified Eagle’s Medium (DMEM; corning, USA) supplemented with 10% heat inactivated FBS, 2 mM l-glutamine ml^−1^, 100 U penicillin ml^−1^, and 100 µg, streptomycin ml^−1^ at 37 °C in a 5% CO_2_ atmosphere.

After harvesting yeast, overnight cultures of yeast strains were suspended with preheated fresh DMEM media and adjusted to O.D 600 nm at 2.0 density (approximately 1 × 10^7^ cfu ml^−1^). One millimeter of each yeast was inoculated to 12-well plates and incubated for 2 h at 37 in a 5% CO_2_ atmosphere. Then, non-adherent yeasts were removed by washing with PBS twice, and the Caco-2 cells and attached yeast were lysed with 1 ml of 0.05% Trypsin–EDTA (Gibco, USA). The adherent yeast was enumerated by diluting the solution serially (1:10) with PBS from the initial and using the drop-plating method on YPD agar^4^.

### Hemolytic activity and biogenic amine production

Safety assessments were conducted by measuring the hemolytic activity and biogenic amine production. The hemolytic activity was evaluated using blood agar plates supplemented with 5% (v/v) defibrinated sheep blood (KisanBio, Korea). The appearance of clear zones around the colonies confirmed by β-hemolysis. After the colony of each strain was streaked on the blood agar, the plates were incubated aerobically at 37 °C for 48 h^57^.

Biogenic amine production was analyzed according to the method of Bover-Cid and Hozapfel^58^. The isolates were streaked on decarboxylase media and incubated aerobically at 37 °C for 4 days. Decarboxylase activity was detected by the color change from yellow to blue.

### ITS region sequencing and phylogenetic analysis

Single colonies were submitted to SolGent Corporation (South Korea) for ITS sequencing. DNA extraction was performed using a boiling method by Chelex bead. The screened and selected strains were identified using amplified internal transcribed spacer ITS 1 (5′-TCC GTA GGT GAA CCT GCG G-3′) and ITS 4 (5′-TCC TCC GCT TAT TGA TAT GC-3′) sequencing^59^. The polymerase-chain-reaction (PCR) reaction was performed in a BigDye® Terminatorv3.1 cycle sequencing kits. Sequencing was analyzed by ABI 3730XL DNA Analyzer (50 cm capillary). The primary measurement (identity, %) was compared to the yeast strain. Sequences were aligned by the NCBI GenBank database using the BLASTn. Phylogenetic analysis proceeded with MEGA software version 11 with neighbor-joining analysis of the ITS region identified *S.cerevisiae* with their type strains. Bootstrap analysis included 1,000 replicates. In addition, sequences were compared with others in the NCBI GenBank database using the BLASTn technique for identification.

### Determination of nitric oxide production

RAW 264.7 cell lines of murine macrophages were obtained from the Korea cell line bank (KCLB, Seoul, Korea). The cells were cultivated in Dulbecco’s modified eagles medium (DMEM, Gibco, USA) supplemented with 10% heat inactivated fetal bovine serum (FBS, Gibco, USA) and 1% antibiotic–antimycotic (Gibco, USA) at 37 ℃ in a 5% CO_2_ atmosphere. RAW 264.7 cells were seeded at 1 × 10^5^ cells ml^−1^ in 24-well plates and stabilized for 2 h. To stabilize overnight cultures of *S. bouladii* CNCM I-745 and *S. cerevisiae* were centrifuged. The cell pellet were washed with PBS twice and adjusted with OD 600 nm at 1.0 density. *S. bouladii* CNCM I-745 and *S. cerevisiae* were heated for 15 min at 110 °C to remove activity of *S. bouladii* CNCM I-745 and *S. cerevisiae*, and then the cells were stimulated with 450 μL of lipopolysaccharides (LPS, 1 μg/mL; Sigma-Aldrich, USA) and 50 μL of heat-killed *S. bouladii* CNCM I-745 and *S. cerevisiae* for 48 h. The incubated cells were centrifuged at 600×*g*, 4 °C for 10 min, and the cell supernatant was transferred to new tubes. The measurement of nitric oxide (NO) concentration was estimated using the Griess reagent (Promega Inc., USA) as the manufacture’s instruction. After mixing the cell supernatant and Griess reagent with the same volumes, the mixture was incubated for 10 min at room temperature, and absorbance at 540 nm was determined by a microplate reader (SpectraMax M4 Microplate/Cuvette Reader, Molecular Devices, USA). The concentration of NO was calculated by comparing it a standard curve^60^.

### In vivo experimental design

Six-week-old female C57BL/6J mice were purchased from Daehan Bio Link Co., Ltd. (Korea). Mice were randomly distributed into 11 groups. After one week of stabilization, the mice were treated with 1.5% dextran sulfate sodium (DSS, MW 36,000–50,000; MP Biomedicals, USA) in distilled water with *S. boulardii* CNCM I-745 or *S. cerevisiae* strain (10^7^ CFU/day) once a day for 14 days, followed by 6 days of recovery (Fig. S2). All mice were subsequently euthanized by carbon dioxide (CO_2_) asphyxiation.

### Colitis evaluation

Mice were examined daily for weight, stool consistency, and total blood in feces for colitis evaluation. DAI was evaluated using the method of Cooper et al.^61^ with minor modification. Scores for weight loss, stool consistency, and bleeding were monitored after 7 days of DSS treatment. After sacrifice, the mouse, colon length and spleen weight per body weight were compared in each group (n = 8).

### RNA isolation, RT-PCR and ELISA

Colon tissue in Buffer RLT was well homogenized. After disruption, the RNA was isolated by RNeasy Plus Mini Kit (Qiagen, Hilden, Germany) protocol. cDNA was synthesized by the PrimeScipt RT reagent Kit (Takara Korea Biomedical Inc.,Seoul, Korea) protocol. Gene amplification was done by the iQ SYBR Green Supermix (Bio-Rad Laboratories, Hercules, CA, USA) protocol. Data were normalized with the housekeeping β-actin expression level. The primers used are listed in Table 4. Serum, feces were quantified by ELISA kits (R&D Systems, Minneapolis, MN, USA) for inflammatory cytokine and myeloperoxidase (MPO), according to the manufacturer’s instructions.

**Table 4:** Gene primer sequences.

Primer	Sequence	References
*β-Actin* (F) *β-Actin* (R)	TCCATCATGAAGTGTGACGT GAGCAATGATCTTGATCTTCAT	Yin et al*.*^62^
*Muc-2* (F) *Muc-2* (R)	GTGCTGCAATATCACCTCATGT TGTATGTGATGGAGCCTGAAAC	Floyd et al.^63^
*ZO-1* (F) *ZO-1* (R)	CCACCTCTGTCCAGCTCTTC CACCGGAGTGATGGTTTTCT	Liu et al.^64^
*Occludin* (F) *Occludin* (R)	CCTCCAATGGCAAAGTGAAT CTCCCCACCTGTCGTGTAGT	
*E-Cadherin* (F) *E-Cadherin* (R)	GCAGTTCTGCCAGAGAAACC TGGATCCAAGATGGTGATGA	Shiohira et al.^65^

### Cell wall β-glucan

β-Glucan (%, w/w) was measured using the yeast glucan assay kit (Megazyme, Ireland). Before the calculation of β-glucan, the yeast cell wall was autolyzed and hydrolyzed following the procedures of Pengkumsri et al*.*^38^. Briefly, yeast cells were incubated in pH 5.0 water at 50 °C for 48 h with shaking at 160 × g, then at 80 °C for 15 min in a water bath. After incubation, yeast cells were harvested by centrifugation for 10 min at 4 °C at 6900×g. The autolyzed yeast cells were mixed with 1.0 M NaOH/HCl and incubated at 80 °C with a stirrer for 2 h. Finally, the hydrolyzed cells’ β-glucan content (%, w/w) was calculated following the assay kit protocol.

### Statistical analyses

The results are the means ± standard deviations of triplicate analyses. Pearson’s correlation and Duncan’s test were performed with SPSS (version 18.0), and results were analyzed to ANOVA using the GraphPad Prism software (GraphPad Software, San Diego, CA, USA).

### Research involving animal participants

For the in vivo experiment, this study was carried out in accordance with the guidelines by the Korean Association for Laboratory Animals, and the protocol was approved by the Institutional Animal Care and Use Committee of Seoul National University (Approval No. SNU-200706-6-2). All studies were performed in compliance with the ARRIVE guidelines.

## Supplementary Information

Supplementary Information.

## Data Availability

The datasets generated during the current study are available in the GenBank (Web link: https://www.ncbi.nlm.nih.gov/genbank/) repository, accession number: OQ247983, OQ247986, OQ248002, OQ248003, OQ248004, OQ248507, and OQ253417. Cell wall β-glucan content of selected *S. cerevisiae* strains and an overview of in vivo screening are available in Supplementary Figs. 1 and 2.
