# Supplementary material for: Probiotic potential of Saccharomyces cerevisiae GILA with alleviating intestinal inflammation in a dextran sulfate sodium induced colitis mouse model
Source: Sci Rep. 2023 Apr 24;13:6687. doi: 10.1038/s41598-023-33958-7 (PMC10125971; doi:10.1038/s41598-023-33958-7)
Supplement: Supplementary file 1 — Supplementary Information. [file 41598_2023_33958_MOESM1_ESM.docx]

**Probiotic potential of *Saccharomyces cerevisiae* GILA** **with alleviating intestinal inflammation in a dextran sulfate sodium induced colitis mouse model**

Bum Ju Kil^1, 2^, Young Jin Pyung^1, 2^, Hyunjoon Park^3^, Jun-Won Kang^4^, Cheol-Heui Yun^1, 2, 3^, and Chul Sung Huh^3,5^*

^1^Biomodulation major, and Center for Food and Bioconvergence, Seoul National University, Seoul 08826, Republic of Korea

^2^Department of Agricultural Biotechnology, and Research Institute of Agriculture and Life Sciences, Seoul National University, Seoul 08826, Republic of Korea

^3^Research Institute of Eco-Friendly Livestock Science, Institute of Green-Bio Science & Technology, Seoul National University, Pyeongchang-gun 25354, Republic of Korea

^4^Department of Food Science and Biotechnology, Dongguk University-Seoul, 32, Dongguk-ro, Ilsandong-gu, Goyang-si, Gyeonggi-do 10326, Republic of Korea

^5^Graduate School of International Agricultural Technology, Seoul National University, Pyeongchang-gun 25354, Republic of Korea

Chul-Sung Huh: chulsunghuh@gmail.com


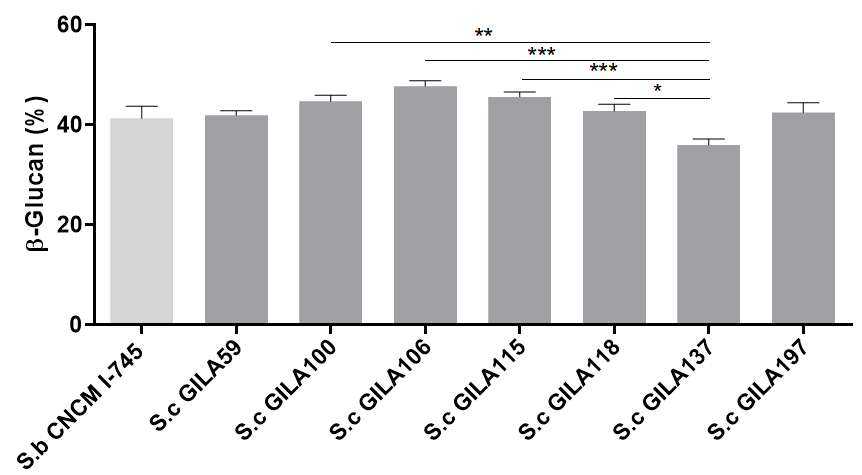


**Supplementary Figure 1.** Cell wall β-glucan content of selected *S. cerevisiae* strains. β-glucan content calculated using a yeast glucan assay kit. The data represent standard errors of the means with duplicates. (**p* <0.05, ***p* <0.01 and ****p* <0.001)


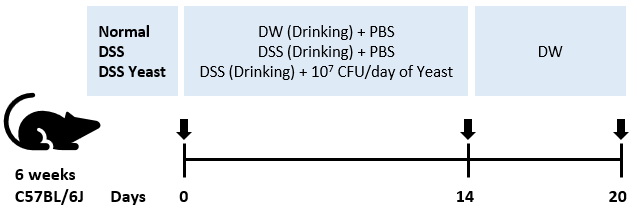


**Supplementary Figure 2.** Overview of *in vivo* screening. Based on Yeast and DSS treatment, these studies include Normal, DSS and DSS with yeast group.
